# Fatigue in Patients with Lung Cancer Is Related with Accelerated Tryptophan Breakdown

**DOI:** 10.1371/journal.pone.0036956

**Published:** 2012-05-16

**Authors:** Katharina Kurz, Michael Fiegl, Bernhard Holzner, Johannes Giesinger, Marianna Pircher, Guenter Weiss, Hubert A. Denz, Dietmar Fuchs

**Affiliations:** 1 Division of Biological Chemistry, Biocenter, Medical University, Innsbruck, Austria; 2 Division of Clinical Immunology and Infectious Diseases, Department of Internal Medicine, Medical University, Innsbruck, Austria; 3 Division of Hematology and Oncology, Department of Internal Medicine, Medical University, Innsbruck, Austria; 4 Department of Psychiatry and Psychotherapy, Medical University, Innsbruck, Austria; 5 Division of Oncology, Hospital Natters, Natters/Innsbruck, Austria; University Hospital of Heidelberg, Germany

## Abstract

**Background:**

Patients with cancer often suffer from fatigue and decreased quality of life which might be related to the breakdown of essential amino acid tryptophan.

**Methods:**

In 50 patients with lung cancer we examined fatigue and the deterioration of quality of life in patients using the Functional Assessment of Cancer Therapy Anemia (FACT-An) and -Fatigue (FACT-F) subscales of FACT-General and the Mental adjustment to Cancer (MAC) questionnaires. Results were compared with tryptophan breakdown as well as serum concentrations of immune activation markers.

**Results:**

Scores of psychological tests correlated significantly with tryptophan breakdown and with circulatory markers of inflammation. However, immune activation and tryptophan breakdown were not related to MAC scores.

**Conclusions:**

Tryptophan breakdown relates with fatigue and impaired quality of life in patients with lung cancer, while declining tryptophan levels are not associated with patients'coping strategies.

## Introduction

Patients with malignant disease often suffer from sustained fatigue and a reduced quality of life (QoL) [1]–[3]. Apart from cancer-related cachexia and tumor-related anemia, additional factors such as anti-tumor chemotherapy contribute to the development of neuropsychiatric complications and deterioration of quality of life (QoL) [4]. Neuropsychiatric symptoms comprise subtle cognitive changes, sleep disturbances, anxiety, but also depression, which strongly affects patients' QoL. Depression is encountered in about 10–25% of cancer patients, a rate that is much higher than in the general population [5]–[7], but similar in chronically ill patients with other medical diagnoses [8]. The prevalence of fatigue in cancer patients is even higher [9], e.g. a study in patients with lung cancer reported about a prevalence of fatigue of 78% [10]. In fact, fatigue is the most commonly reported symptom in cancer patients and greatly affects their QoL [9]. The feeling of tiredness and lack of energy appear to result from a multifactorial etiology, both physical and psychological components play a role [11], [12]. Anemia is considered to be a main factor causing fatigue [13], [14], but also other factors like dyspnoea, non-refreshing sleep and depression can contribute [15]–[17]. In addition, immune activation has been proposed to induce fatigue and depression in patients with cancer or other chronic diseases [17]–[22].

The development of behavioral symptoms has been attributed to increased concentrations of pro-inflammatory cytokines [21]–[23]. Thereby an increased catabolism of the essential amino acid L-tryptophan, which is a precursor of the neurotransmitter serotonin, has been suggested to play a major role in this setting [24]–[26]. The enzyme indoleamine 2,3-dioxygenase (IDO) converts tryptophan to kynurenine preferentially upon stimulation with pro-inflammatory cytokines like interferon-γ (IFN-γ) [18], [27]–[29]. In parallel, IFN-γ induces the enzyme GTP-cyclohydrolase I in human macrophages to form and release neopterin [27], [30].

Enhanced activation of IDO in parallel with increased neopterin formation has been described in patients with hematological malignancies [31], colorectal cancer [32], [33], gynecological malignancies [34] and also in other malignant diseases [35]. Interestingly, enhanced tryptophan breakdown was found to be related to an impaired QoL in patients suffering from colorectal cancer [33], and it was also predictive for an increased fatigue feeling and worse QoL in a population of patients with different types of cancer [35]. As these studies suggest that immune-mediated tryptophan breakdown might play a role in the development of fatigue and might also be involved in the impairment of QoL in patients suffering from lung cancer, we wanted to better characterize the relationship between concentrations of immune activation markers and QoL and fatigue by using well-established and validated questionnaires and also self-assessment scores of patients. Furthermore, we also investigated whether coping strategies and physical performance of patients are related to tryptophan breakdown and immune activation.

## Methods

### Participants

Fifty patients suffering from lung cancer were recruited from the district hospital of Natters near Innsbruck/the Tyrol. Patients' characteristics as well as their concomitant medication and presence/absence of infection are shown in Table 1.

**Table 1:** Baseline characteristics of patients.

50 subjects (37 men, 13 women), median age 65 years	
**Type of lung cancer:** Non small lung cell cancer (NSCLC; n = 38), Small lung cell cancer (SLCC, n = 12) 38 (%)	
**Tumor stage of patients:** UICC Stage I (n = 4), stage II (n = 8), stage III (n = 16), stage IV (n = 22)	
Smokers and former smokers (n)	40 (80%)
Patients with infection (n)	9 (18%)
Patients who survived the following 3 months (n)	40
**Concomitant medication of patients:**	
Chemotherapy (n)	36
Radiotherapy (n)	8
Antidepressant medication (n)	21
Morphin treatment (n)	5
Anti-obstructive treatment (n)	31
Proton pump inhibitor (n)	37
Analgesic therapy (n)	26
Anti-infective therapy (n)	9
Antihypertensive medication (n)	21
Cardiovascular medication (n)	26
Thyroid medication (n)	8
Uricosurics	9

Within the scope of routine blood examinations, fractions of serum samples of patients were collected and frozen at −20°C until analysis. To assess patients' performance status, the ECOG scala (Eastern Cooperative Oncology Group) was used.

The study was approved by the ethical committee of the Innsbruck Medical University and patients gave written informed consent to participate in the study. All clinical investigation has been conducted according to the principles expressed in the Declaration of Helsinki.

### Psychometric Tests

#### FACT-anemia and FACT-fatigue subscale, MAC

To assess fatigue of patients, the fatigue subscale of FACT-G (FACT-F) and the Anemia subscale of FACT-G questionnaire (FACT-An), respectively, were employed [36]. The FACT-F is a 13-item fatigue subscale utilizing a five-point Likert self-report scale ranging from 0 (not at all) to 4 (very much). After accounting for reverse-scored items, answers are summed across the subscales and added for a total score, with higher scores indicative of less fatigue feeling. The total score varies from 0 (worst condition) to 52 (best condition). The FACT-An subscale is a questionnaire assessing fatigue and anemia-related concerns in patients with cancer and comprises questions of the 13-item fatigue subscale and seven questions specifically investigating non–fatigue-based anemia symptoms. Scores range from 0 to 80, with higher scores representing better functioning and well-being of patients.

Patients also completed MAC questionnaires (Mental adjustment to Cancer) to assess their coping strategies [37]. This 40-items questionnaire includes five subscales focusing on responses to being diagnosed with cancer. Items are rated on a scale ranging from “definitely does not apply to me” ***(1)*** to “definitely applies to me” ***(4)***. The fighting spirit (FS) subscale assesses whether the patient views cancer as a challenge and takes an active and optimistic role in his or her treatment; the helplessness-hopelessness (H) subscale assesses whether the patient has an attitude of uncontrollability and hopelessness toward cancer and its outcome; the anxious preoccupation (AP) subscale assesses whether the patient has an overly anxious and diffuse preoccupation with cancer; and the fatalism-stoic acceptance (F) subscale assesses whether the patient exhibits a passive, fatalistic, and stoic acceptance of cancer. To assess the general attitude of patients towards their disease (rather positive or negative adjustment), overall scores were calculated: the Summary Positive Adjustment (SPA) Scale and the Summary Negative Adjustment (SNA) Scale [38].

#### Self-assessment of patients

In addition to the FACT-scores we also asked patients to assess their fatigue feeling on a scale from 1–5 (patients' self-report: 1 = no fatigue; 5 = high grade of fatigue), independently of a questionnaire. Patients furthermore scored their QoL on the same scale from 1–5 (patients' self-report: 1 = best score, very good; 5 = worst score, very bad). The purpose of this self-assessment was to compare the patients' view with results of the FACT-questionnaires.

### Measurements

Serum concentrations of tryptophan and kynurenine were measured by high performance liquid chromatography as described [39]. After precipitation of protein with trichloroacetic acid, tryptophan was measured by fluorescence detection at 285 nm excitation and 365 nm emission wavelengths. Kynurenine was monitored by UV-absorption at 360 nm wavelength. L-nitrotyrosine was used as an internal standard, and standard preparations containing tryptophan, kynurenine and nitrotyrosine in the presence of albumin underwent the whole procedure like serum specimens. To estimate IDO activity, the ratio of the concentrations of the enzyme product kynurenine to the substrate tryptophan (kynurenine to tryptophan ratio = Kyn/Trp) was calculated [40].

Neopterin concentrations were determined by ELISA (BRAHMS Diagnostica, Hennigsdorf, Germany), and C-reactive protein (CRP) concentrations were determined with Ektachem Clinical Chemistry Slides according to instructions of the manufacturers. Blood sedimentation rate was measured according to Westergren.

### Statistical Analyses

As not all data sets showed normal distribution, non-parametric tests were employed (Kruskal-Wallis, Mann-Whitney U-test, two-sided tests). Spearman rank correlation analysis was applied to assess associations between variables, partial correlation analysis was employed to adjust for confounders like tumor progression, age, gender or treatment status. To account for multiple testing Bonferroni correction was applied, i.e. only p-values <0.01 were regarded as significant. Univariate binary logistic regression analysis (inclusion method) was used to identify parameters indicative for survival and fatigue. Multivariate linear regression analysis was performed to further investigate predictors of psychological outcomes (FACT-F/FACT-An and MAC). Variables included in forward predictor selection were clinical and sociodemographic variables as well as blood parameters. P–values <0.05 were considered to indicate statistical significance.

To assess the internal consistency of the used questionnaires and subscales, Cronbach's alpha coefficients were calculated.

## Results

### Psychological Outcomes

Patients with lung cancer had a mean FACT-An score of 51.2±2.3 (mean ± S.E.M.) and a mean FACT-F score of 31.3±1.9. According to the FACT-F subscale, seven patients suffered from very severe (scores 0–14) and 7 more patients from severe fatigue (scores 15–24). Eleven patients complained about moderate fatigue (scores 25–34), while 14 patients reported about little (FACT-F scores 35–44) and 11 about no fatigue (FACT-F scores >45). The internal consistency of the used fatigue questionnaires was very good (Cronbach's alpha for FACT-F subscale was .9247, Cronbach's alpha for FACT-An subscale was .9214).

When patients scored their fatigue on a self-assessment scale from 1–5 (1 = no fatigue; 5 = high grade of fatigue), a mean score of 2.6 (±.1) was achieved. One patient reported about very severe fatigue, 4 patients about severe fatigue and 22 patients about moderate fatigue. One patient did not feel fatigued at all, while 21 reported about little fatigue.

Patients' self-assessment of their QoL (with 1 = best score, very good; 5 = worst score, very bad) showed a mean score of 2.7 (±.1). One patient assessed his QoL as very good, while 9 patients reported about a bad QoL; 21 patients assessed their QoL as satisfying, while 19 patients expressed that their QoL was moderately impaired.

Performance status of patients according to ECOG criteria was 3.0 in the mean (±.1), 16 patients had an ECOG performance status of 2, i.e. they were ambulatory and able to care for themselves, but not able to work anymore, while 17 were only capable of limited self-care (EGOG score 3), and 17 were completely disabled (ECOG score 4) and thus had to be cared for by others.

Mean MAC scores for “Fighting spirit” were 48.9±1.0 (maximum score: 64), 22.1±.6 for “Anxious preoccupation” (maximum score: 36), 11.9±.6 for “Hopelessness” (maximum score: 24), 1.5±.2 for “Avoidance” (maximum score: 4) and the mean score for Fatalism was 2.8±.5 (maximum score: 32). According to these scores patients had a strong fighting spirit, but also tended to be fatalistic and anxious. To assess the general attitude of patients towards their disease (rather positive or negative adjustment), also overall scores were calculated: the Summary Positive Adjustment (SPA) Scale was 52.2±1.1 and the Summary Negative Adjustment (SNA) Scale was 35.6±1.2. The internal consistency of SPA and SNA scales was higher than that of the original five subscales (Cronbach's alpha for SPA was .8002 and .8126 for SNA Scale), Cronbach's alpha coefficients varied from .3747 (MAC-Fatalism) to .7819 (MAC-Hopelessness).

Patients who died from cancer within 3 months had lower FACT- F (mean ± SEM 23±4 *vs.* 33±2, p<0.01) and FACT-An scores (mean ± SEM 39±5, *vs.* 51±3; p<0.01) than survivors, while self-assessment scores regarding fatigue and QoL and MAC scores did not differ.

Patients under treatment with antidepressants (n = 21) had lower FACT-An and FACT-F scores than patients without antidepressant medication (both p<0.05), but higher MAC-FS and MAC-H scores (both p<0.05). No differences regarding psychological scores were seen between patients with NSCLC and SCLC, as well as between patients with higher or lower tumor stage.

### Relationship between Different Psychological Tests

To assess the psychological situation of patients with lung cancer, overall scores for FACT-An, FACT-F and MAC were calculated. These scores as well as the patients' self-assessment scores for fatigue and QoL were correlated with each other.

Significant correlations were seen between fatigue scores (FACT-F, FACT-An) and patients' self-assessment of fatigue feeling and their QoL. Spearman rank coefficients for fatigue self-assessment and FACT-F and FACT-An were rs = −0.547 for FACT-F and rs = −0.537 for FACT-An (both p<0.001). Also patients' assessment of their QoL was strongly related to FACT-scores: Spearman rank coefficients for QoL and FACT-F and FACT-An were rs = −0.466 for FACT-F and rs = −0.494 for FACT-An, respectively (all p<0.001). Patients who reported stronger fatigue mostly also claimed to experience a reduced QoL (rs = 0.663, p<0.001).

Patients with higher MAC- scores for “Fighting spirit” reported about less fatigue (rs = 0.489 for FACT-F and rs = 0.521 for FACT-An; both p<0.001), whereas higher MAC-scores for “Hopelessness” coincided with more fatigue (rs = −0.419 for FACT-F and rs = −0.424 for FACT-An; all p<0.01). Positive adjustment to cancer scores were associated with higher FACT-F and FACT-An scores (i.e., less fatigue feeling; rs = 0.441 for FACT-F and rs = 0.570 for FACT-An; both p<0.001), while negative adjustment to cancer was correlated with a stronger fatigue feeling (rs = −0.429 for FACT-F and rs = −0.420 for FACT-An; both p<0.01).

### Tryptophan Metabolism, Immune Activation, Anemia and Tumor Stage

**Figure 1:**
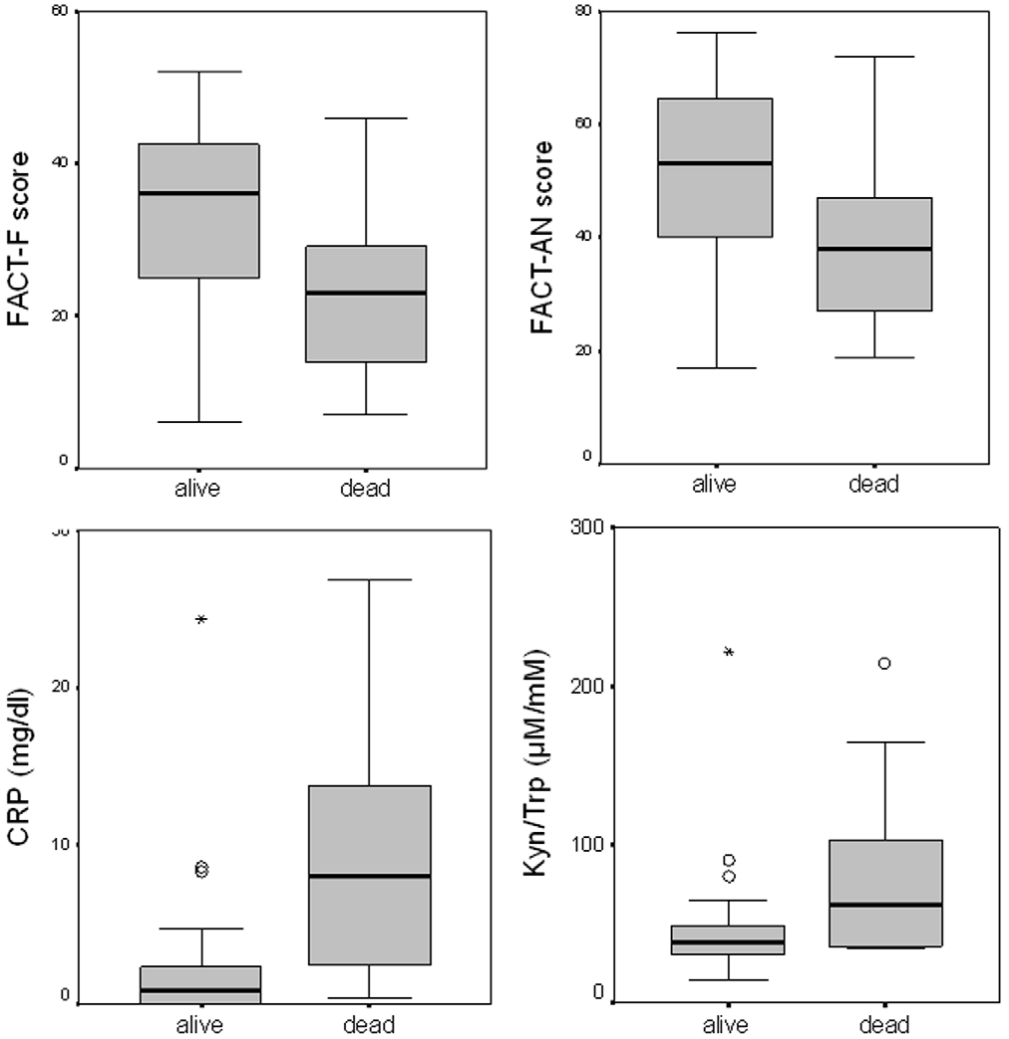
FACT-F (upper left) and FACT-AN scores (upper right) as well as concentrations of C-reactive protein (CRP; lower left) and kynurenine to tryptophan ratios (Kyn/Trp; lower right) of patients with lung cancer who have died (n = 10) or were still alive or after 3 months of follow-up (all group comparisons between alive and dead p<0.01).

The mean tryptophan concentration in patients was 53.4±2.3 µmol/L (mean ± S.E.M.), mean kynurenine concentration was 2.4±.1 µmol/L and the mean Kyn/Trp was 52.3±6.0 µmol/mmol. In comparison to the ranges observed in healthy controls (35), the cancer patients presented with lower tryptophan and higher kynurenine levels, and accordingly with increased Kyn/Trp. Higher tryptophan levels and lower Kyn/Trp were observed in patients who survived the following 3 months of observation (both p<0.01, see also Fig. 1).

Patients with SCLC showed similar tryptophan and kynurenine concentrations as patients with NSCLC, but were significantly younger (p<0.05). No differences regarding tryptophan metabolism were seen between patients with advanced tumor stages (UICC-stage 3 or 4) and those with earlier tumor stages.

Concentrations of inflammatory and immune activation markers were also increased in comparison to reference ranges (neopterin: 12.3±1.4 nmol/L, CRP: 4.07±.98 mg/dl).

Patients who died within 3 months had higher CRP levels than survivors (p<0.01), while neopterin concentrations did not differ significantly. Interestingly, neither neopterin nor CRP concentrations were associated with tumor stage in our population.

Mean hemoglobin concentrations in patients was 13.2±0.3 mg/dl, 18 patients (15 men, 3 women) suffered from anemia with hemoglobin concentrations <12 mg/dl for women and <13 mg/dl for men). Hemoglobin levels were lower in patients with higher tumor stage (rs = −.393, p<0.01), and the majority of the anemic patients (n = 11, 61.1%) had a more progressed tumor disease (UICC-stage III or IV). Patients who died within the next 3 months of observation had lower hemoglobin levels than patients who survived (p<0.01). Lower hemoglobin levels were also predictive for death within the next 3 months of observation. Patients with anemia (n = 18) presented with higher inflammation and immune activation markers (CRP: 7.3±1.9 *vs.* 1.3±.4 mg/dl, p<0.001; neopterin: 16.6±3.2 nmol/L *vs.* 9.0±1.0 nmol/L, p<0.05) and lower tryptophan levels (42.9±3.0 µmol/L *vs.* 59.0±2.9 µmol/L), and accordingly with an increased Kyn/Trp (65.6±11.6 *vs.* 45.8±7.3 µmol/mmol) as compared to cancer patients without anemia

Enhanced tryptophan degradation coincided with immune activation: Significant associations were seen between inflammatory markers and tryptophan catabolism: Patients with high neopterin concentrations had higher kynurenine levels (rs = 0.410, p<0.01), a higher Kyn/Trp (rs = 0.556, p<0.001) and higher CRP concentrations (rs = 0.558, p<0.001). CRP levels correlated with tryptophan concentrations rs = −0.468, p = 0.001) and Kyn/Trp (rs = 0.488, p<0.001), indicating that tryptophan degradation was related to inflammation.

Inflammation and tryptophan degradation were both associated with decreased hemoglobin values: Significant associations existed between hemoglobin concentrations and CRP (rs = −0.563, p<0.001), Kyn/Trp (rs = −0.416, p<0.01) as well as tryptophan levels (rs = 0.533, p<0.001).

### Relationship between Psychological Scores and Tryptophan Breakdown

Patients with moderate to severe fatigue according to FACT-F scores (score 0–34, n = 25) presented with higher concentrations of inflammation markers, a higher degree of tryptophan breakdown and lower hemoglobin levels than patients with little or no fatigue according to their FACT-F scores (score >34; n = 25; see Table 2). Furthermore, they reported about a worse QoL (p<0.05) and had worse ECOG scores (p<0.001, Table 2).

**Table 2:** Mean concentrations (± SEM) of investigated lab parameters and psychological scores of lung cancer patients with moderate to severe and little or no fatigue, respectively (n.s. = not significant).

	Patients with moderate to severe fatigue (n = 25)	Patients with little or no fatigue (n = 25)	p-value
Hemoglobin (mg/dl)	12.5±0.4	13.8±0.4	p<0.05
CRP (mg/dl)	6.25±1.74	2.33±0.99	p<0.05
Neopterin (nM)	14.5±2.1	10.0±1.6	p<0.05
Tryptophan (µM)	49.6±2.9	57.2±3.4	n.s.
Kynurenine (µM)	2.6±0.2	2.2±0.2	n.s.
Kyn/Trp (µM/mM)	60.7±9.1	43.9±7.5	p<0.05
Leukocytes (/µl)	10.1±1.3	8.8±0.9	p<0.05
Quality of life	3.0±0.2	2.5±0.1	p<0.05
Fatigue	3.0±0.2	2.3±0.1	p<0.001
Fighting spirit	45.9±1.27	51.8±1.23	p<0.01
Anxious preoccupation	22.9±0.90	21.3±0.85	n.s.
Fatalism	21.2±0.66	20.4±0.77	n.s.
Hopelessness	13.4±0.91	10.3±0.56	p<0.05
Avoidance	1.68±0.18	2.2±0.24	n.s.
ECOG score	3.4±0.1	2.5±0.1	p<0.001

**Figure 2:**
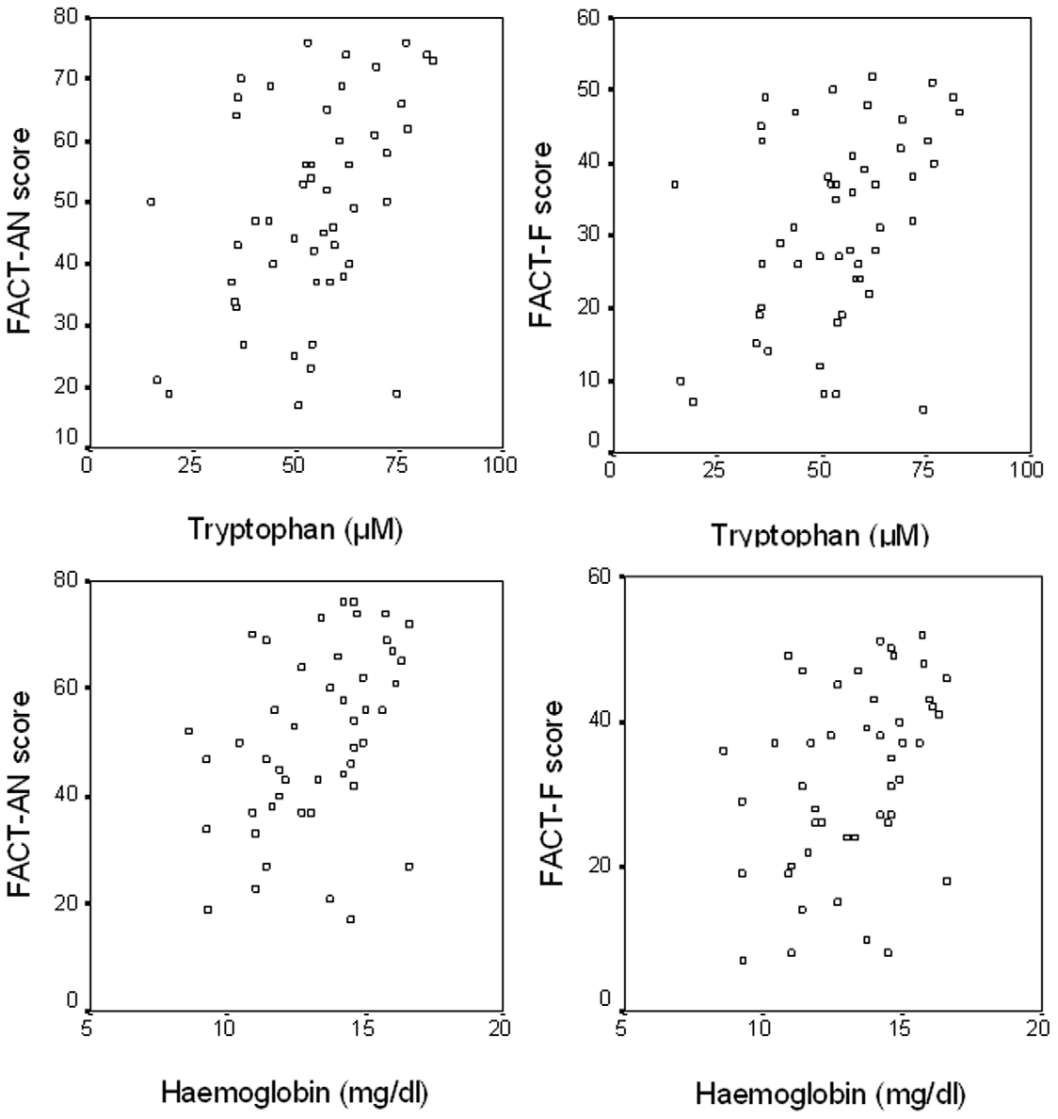
Correlations of FACT-AN and FACT-F- scores with tryptophan and hemoglobin concentrations: tryptophan *vs.* FACT-AN (upper left): rs = 0.409, p<0.01; tryptophan *vs.* FACT-F (upper right): rs = 0.376, p<0.01; hemoglobin *vs.* FACT-AN (lower left): rs = 0.426, p<0.01; hemoglobin *vs.* FACT-F (lower right): rs = 0.400, p<0.01.

**Figure 3:**
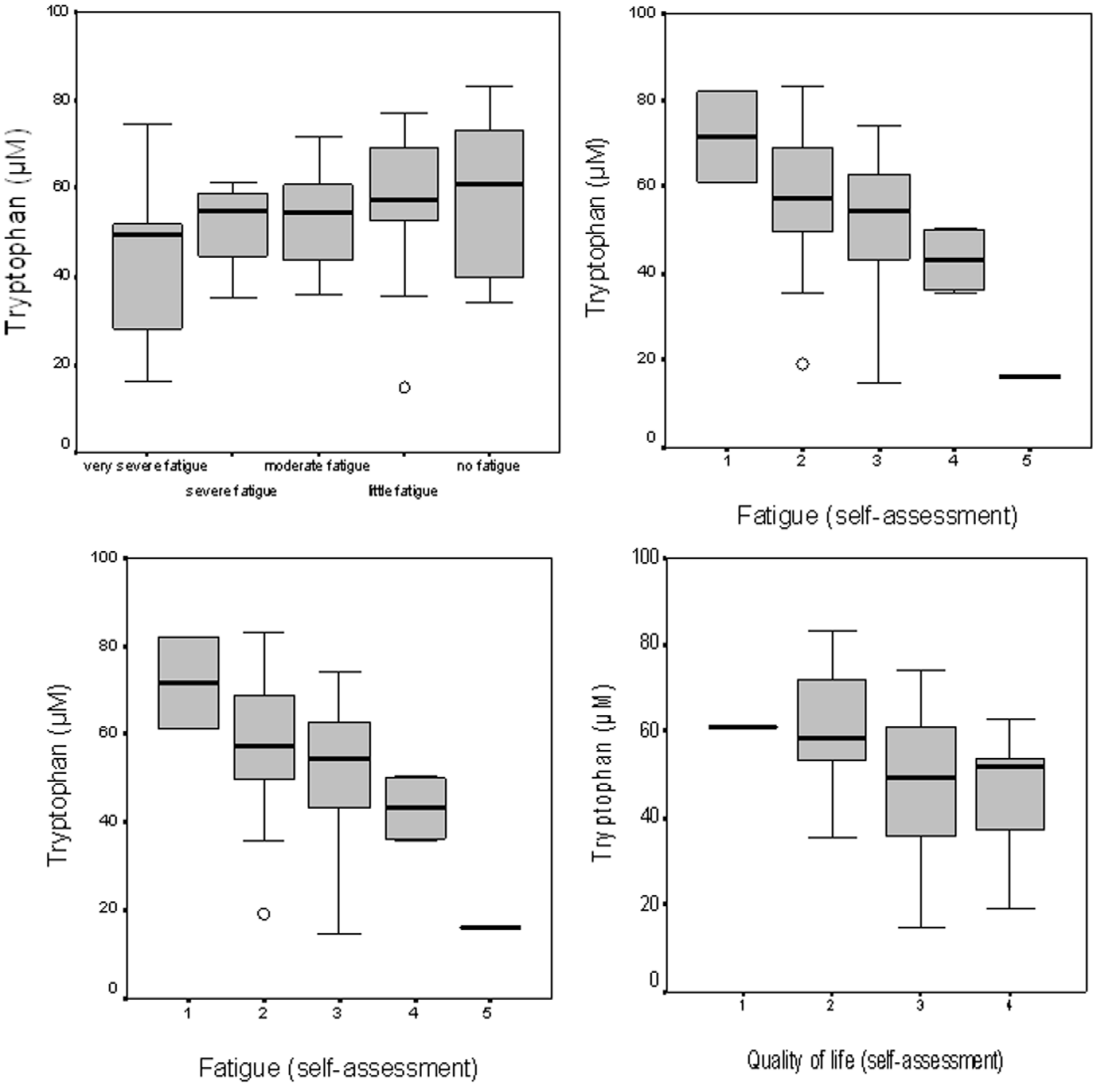
Tryptophan concentrations are related with fatigue feeling in patients according to their FACT-F score (upper left), patients' self-assessment of their fatigue (upper right), patients' physical performance (ECOG score; lower left), and patients' self-assessment of their quality of life (lower right).

Significant associations existed between FACT-An, FACT-F scores and markers of immune activation and tryptophan catabolism as detailed in Table 3 and shown in Figures 2 and 3. Interestingly, significant correlations were only found in patients without antidepressant treatment, while associations between fatigue scores and immune activation and tryptophan breakdown did not exist in patients under antidepressant treatment.

**Table 3:** Spearman rank correlations (two-sided) between investigated parameters of immune activation, haemoglobin levels and fatigue scores as well as patients' self-assessment scores and ECOG scores.

	FACT-F score	FACT-An score	Quality of life	Fatigue	ECOG score
**Neopterin (nM)**	−0.412 *	−0.412 *	0.181	0.178	0.378 *
	p = 0.003	p = 0.003	n.s.	n.s.	p = 0.007
**Tryptophan (µM)**	0.376 *	0.409 *	−0.382 *	−0.347	−0.277
	p = 0.007	p = 0.003	p = 0.006	p = 0.01	p = 0.05
**Kynurenine (µM)**	−0.123	−0.112	−0.104	0.113	−0.238
	n.s.	n.s.	n.s.	n.s.	p = 0.09
**Kyn/Trp (µM/mM)**	−0.323	−0.329	0.146	0.232	0.376 *
	p = 0.02	p = 0.02	n.s.	n.s.	p = 0.007
**CRP (mg/dl)**	−0.459 *	−0.468 *	0.219	0.226	0.310
	p = 0.002	p = 0.001	n.s.	n.s.	p = 0.04
**Hemoglobin (mg/dl)**	0.400 *	0.426 *	−0.312	−0.223	−0.339
	p = 0.005	p = 0.003	p = 0.03	n.s.	p = 0.02

Only p-values ≤0.01 were regarded as significant (after Bonferroni correction for multiple testing). Significant correlations are indicated by asterisks.

Also performance status of patients and self-assessment of their QoL were correlated with tryptophan concentrations (see also Figure 3). Similarly, hemoglobin levels were associated with fatigue scores (rs = 0.400 for FACT-F, rs = 0.426 for FACT-An, both p<0.01, Table 3 and Fig. 2).

Hemoglobin and CRP levels were predictive for the presence of moderate to severe fatigue in binary logistic regression analysis (hemoglobin: OR 0.716 [0.513–0.998]; CRP: OR 1.177 [1.033–1.341], both p<0.05).

None of the MAC-scores correlated with laboratory markers of immune activation, tryptophan breakdown or hemoglobin levels. ECOG scores were higher in patients with lower fighting spirit (rs = −0.519, p<0.001) and with higher hopelessness scores (rs = 0.378, p<0.01) as well as in patients with lower scores of positive adjustment to cancer (rs = −0.544, p<0.001).

Multivariate regression analysis was performed to further investigate predictors of psychological outcomes (FACT-F/An and MAC). Variables included in forward predictor selection were clinical and sociodemographic variables as well as blood parameters. In addition to bivariate associations shown in the correlation analyses above, we found multivariate predictor sets for FACT-F (CRP and antidepressant intake; explained variance 26.3%; p = 0.001) and MAC-Fatalism (sex, age, tumor stage; explained variance 28.9%; p<0.001).

## Discussion

A high percentage of the patients with bronchus carcinoma (50%) suffered from moderate to severe fatigue according to FACT-F scores. Patients with fatigue acclaimed a significantly worse QoL and performed worse than those with little or no fatigue, which is well in line with earlier studies [1]–[3]. The self-assessment of patients regarding their fatigue showed a good correlation with results of the FACT-F and FACT-An scores indicating that results of self-assessment scores (which are gained by a simple question) are similar to and even comparable to results of established questionnaires, which are more time-consuming, but can characterize patients' fatigue feeling more explicitly.

Patients who suffered from a higher degree of fatigue presented with lower tryptophan concentrations and a higher degree of immune activation. Higher Kyn/Trp and lower tryptophan concentrations in parallel with higher concentrations of inflammation markers were also observed in patients who performed worse according to their ECOG score and in patients with anemia.

Anemia is supposed to be one of the important triggers of fatigue, and immune activation and inflammation are well established to play a major role in the development of anemia of chronic disease (ACD) [14], [41]. Studies in patients with cancer and also HIV infection have demonstrated correlations between neopterin and hemoglobin levels [31], [35], [41]–[43]. Enhanced tryptophan breakdown in parallel with higher neopterin levels has been observed in patients with ACD [44], indicating that enhanced tryptophan catabolism and as a consequence, tryptophan depletion, might affect hematopoiesis. Significant correlations between hemoglobin and tryptophan concentrations on the one hand, and elevated Kyn/Trp on the other hand in our patients would be well in line with this hypothesis. Anemia patients showed a higher degree of tryptophan breakdown than patients with normal hemoglobin concentrations. More than one third of our patients (36%) were anemic, and anemia was a significant predictor of fatigue. However as already shown previously [45], anemia is prominently involved, but certainly not the only factor in the pathogenesis of cancer-related fatigue. Fatigue and also reduced physical performance are among other symptoms frequently encountered in patients with depression [46].

Interestingly, associations between immune-mediated tryptophan breakdown and fatigue were only significant in patients without antidepressant medication, which fits well with results of an earlier study in HIV-infected patients [47]. In that study, correlations between immune activation and Becks depression score as well as QoL Score MQoL-HIV were encountered only in patients without antidepressant treatment. Our data thus indirectly indicate that immune activation and tryptophan breakdown might be related with the development of fatigue. Antidepressant treatment appeared to influence the relationship between fatigue feeling and the biochemical pathways we investigated in our population of lung cancer patients: Patients under antidepressant treatment had lower tryptophan and higher CRP levels than those without this medication, also indicating indirectly that enhanced tryptophan breakdown might be involved in the development of depression. However, this finding may be biased by the fact that we had only 50 patients and a high percentage of patients with antidepressant medication.

Tryptophan is the precursor of neurotransmitter serotonin, and thus, increased tryptophan breakdown might lead to decreased serotonin formation or trigger depressive-like behaviour by the accumulation of neurotoxic metabolites of kynurenine: Elevated concentrations of neurotoxins quinolinic acid and 3-hydroxy-kynurenine have been shown in the CSF and also brains of patients with inflammatory neurological diseases [48]. Quinolinic acid interferes with the NMDA receptor and alleviates NMDA-mediated induction of apoptosis of primary neuronal cell cultures, while 3-hydroxy-kynurenine generates free radicals, which can cause oxidative stress and lipid peroxidation [49]. Kynurenic acid on the other hand is an endogenous neuroprotect [50], the formation of which is increased in patients with inflammatory neurological diseases [48], but in fact, the balance between neurotoxic and neuroprotective kynurenine metabolites seems to be shifted towards neurotoxins in patients with chronic immune activation [51], [52] and also in patients with major psychiatric disorders [53].

Also a recent study in mice points to a crucial role of IDO in the development of depressive-like behaviour: Chronic infection with BCG induced depressive-like behaviour in normal mice, while IDO-deficient mice were resistant to BCG-induced depressive-like behaviour [54]. Interestingly, IDO-deficient mice showed a normal induction of pro-inflammatory cytokines in response to BCG infection, supporting the concept of a central role of IDO and tryptophan in the development of depression. However, there is also the possibility that tryptophan catabolism can be stress-induced when hepatic tryptophan 2,3-dioxygenase (TDO) is upregulated. In fact, IDO expression in cells or tissues was not examined in our cancer patients, but the significant relationship found between neopterin and Kyn/Trp supports a role of IDO in the cytokine-induced tryptophan metabolic changes observed in our patients.

Recent data by Capuron and coworkers are in line with this hypothesis, in fact increased tryptophan catabolism was associated with the depressive symptoms of lassitude, reduced motivation, anorexia, and pessimism, while disturbed phenylalanine/tyrosine/dopamine metabolism was shown to be related with neurovegetative symptoms, like sleep disturbance, digestive symptoms, sickness, motor symptoms and fatigue in elderly persons [55].

As one would expect patients with fatigue had higher hopelessness scores and less fighting spirit, and their positive adjustment to cancer was worse. MAC scores were not correlated with immune activation or tryptophan catabolism, and were also not predictive for the survival of patients. Patients' attitude towards their disease might influence both, their fatigue feeling and also their immune activation status, however, from our data we cannot draw conclusions regarding this relationship- in fact, it could also be the other way round.

Patients with higher fatalism score were those with more progressed tumor stage, physical performance according to their ECOG score was better in patients with higher fighting spirit and with low hopelessness scores. It would be interesting to know whether inflammation-induced noradrenergic disturbances [56] are associated to this spectrum of symptoms. Also the question, whether psychological intervention might be effective to interfere with immune-mediated tryptophan degradation, appears to be worth further examination- as psychological intervention was shown to alleviate depressive symptoms [57].

There are several limitations of this study: The cohort studied is quite heterogeneous with different types of lung cancer, different stages and different treatment regimes including antidepressants and chemotherapy. However, the analysis of the various subgroups would be underpowered because of the still small size of the study population. As highlighted in the introduction section, sleep disturbances might relate to the development of fatigue and depression in patients [15]–[17], but no validated sleep quality questionnaire such as the Pittsburgh Sleep Quality Index has been performed. Thus, the relationship between the metabolic changes found could not be analyzed. Also no direct measure of the enzyme pathways of tryptophan metabolism has been performed to further support the potential roles of IDO vs. and TDO. Finally this cross-sectional study can only provide correlational evidence but cannot prove any cause-effect relationship.

In conclusion, this study confirms that fatigue is frequently encountered in patients with lung cancer and indicates that tryptophan breakdown might play a role in the development of fatigue and probably also depression in these patients. Still, it has to be kept in mind that our population of lung cancer patients was quite small, and that results are therefore rather preliminary. Further longitudinal studies with more patients with cancer are therefore needed to enable a better understanding of underlying biochemical processes. They may also be able to provide new information, as to whether therapeutic modulation of tryptophan availability may affect fatigue and depression in patients with cancer.
